# Impact of Lutein Supplementation on Liver Health, Oxidative Metabolism, Gut Microbiota, and Growth Performance in High-Fat Diet Fed Mice

**DOI:** 10.3390/nu18152414

**Published:** 2026-07-24

**Authors:** Xiaojing Wu, Abdur Rahman, Hairui Yu, Muhammad Uzair Akhtar, Chengyu Ma, Jiayi Zhang, Leyong Yu, Lingyao Li, Govindhrajan Sattanathan, Shahid Sherzada, Baobin Lu

**Affiliations:** 1 College of Fisheries and Life Science, Shanghai Ocean University, Shanghai 201306, China; wuxiaojing2023@163.com (X.W.); mcy3199268616@163.com (C.M.); lubaobin183@163.com (B.L.); 2 Key Laboratory of Biochemistry and Molecular Biology in Universities of Shandong, Weifang Key Laboratory of Coho Salmon Culturing Facility Engineering, Institute of Modern Facility Fisheries, College of Advanced Agriculture and Life Sciences, Weifang University, Weifang 261061, China; sattanathanphd@gmail.com; 3 Shandong Freshwater Fisheries Research Institute, Jinan 250013, China; 4 Department of Animal Sciences, University of Veterinary and Animal Sciences, Lahore 54000, Pakistan; 5 Department of Animal Nutrition, Cholistan University of Veterinary and Animal Sciences, Bahawalpur 63100, Pakistan; 6 Department of Physiology and Pharmacology, Karolinska Institutet, 17177 Stockholm, Sweden; zhangjiayiazan1005@163.com; 7 College of Fisheries, Huazhong Agricultural University, Wuhan 430070, China; leyong618@gmail.com; 8 Shandong Collaborative Innovation Center of Coho Salmon Health Culture Engineering Technology, Shandong Conqueren Marine Technology Co., Ltd., Weifang 261108, China; 980714742@163.com; 9 Weifang Key Laboratory of Salmon and Trout Health Culture, Conqueren Leading Fresh Science and Technology Inc., Ltd., Weifang 261205, China; 10 Saveetha Medical College and Hospital, Saveetha Institute of Medical and Technical Sciences, Saveetha University, Thandalam, Chennai 602105, India; 11 Department of Zoology, Government College University, Lahore 54000, Pakistan; shahid.sherzada@gcu.edu.pk

**Keywords:** antioxidant, gut microbiota, lipid metabolism, metabolic disorder, lutein

## Abstract

**Background/Objectives**: Natural bioactive compounds are promising interventions for obesity-related metabolic dysfunction. Lutein, a xanthophyll carotenoid with antioxidant and anti-inflammatory properties, may improve metabolic health, but its effects on liver function and gut microbiota in high-fat diet (HFD)-induced obesity are not fully elucidated. This study evaluated the effects of lutein supplementation on hepatic function, oxidative status, lipid metabolism, and gut microbiota composition in HFD-fed mice. **Methods**: Male C57BL/6J mice (6 weeks old) were randomly assigned to four groups (*n* = 8/group): Ctrl (low-fat diet), model (HFD), low-dose lutein (50 mg/kg/day), and high-dose lutein (100 mg/kg/day). **Results**: Following a 70-day intervention, lutein supplementation, particularly at the higher dose, reduced the liver index, improved serum lipid profiles, liver injury markers, and hepatic antioxidant capacity by increasing superoxide dismutase, catalase, and glutathione levels while reducing malondialdehyde content, and alleviated hepatic histopathological alterations. Lutein supplementation was also linked to elevated gut microbial richness and shifts in the relative frequency of several bacterial taxa, including partial restoration of *Bacteroidota* and enrichment of *Roseburia* and *Faecalibaculum*. These findings demonstrate that lutein supplementation attenuated HFD-induced metabolic and hepatic alterations, accompanied by improved antioxidant status and modulation of gut microbiota. **Conclusions**: Overall, lutein alleviated hepatic steatosis, oxidative stress, and metabolic dysfunction, highlighting its potential as a dietary strategy for managing obesity-related liver functions and gut dysbiosis.

## 1. Introduction

As a liver-specific manifestation of metabolic syndrome, metabolic-associated fatty liver disease (MAFLD) has become one of the most prevalent chronic liver diseases worldwide, with its incidence increasing in parallel with the global obesity epidemic [1]. The MAFLD is characterized by excessive lipid accumulation in hepatocytes accompanied by insulin resistance, dysregulated lipid metabolism, oxidative stress, and chronic low-grade inflammation, and it may further progress to steatohepatitis, fibrosis, cirrhosis, and even hepatocellular carcinoma [2]. Increasing evidence indicates that the pathogenesis of MAFLD involves multiple parallel mechanisms, including lipotoxicity, mitochondrial dysfunction, endoplasmic reticulum stress, oxidative stress, and gut-derived inflammatory signals [3,4,5]. These pathological alterations not only impair hepatic function but also contribute to systemic metabolic abnormalities associated with obesity [6,7].

Accumulating evidence suggests that the gut microbiota plays an essential role in regulating host metabolic homeostasis and is closely associated with the development and progression of obesity-related liver diseases [8]. High-fat diet (HFD)-induced gut dysbiosis can increase intestinal permeability, promote the translocation of microbial products into the portal circulation, and exacerbate hepatic inflammation and metabolic dysfunction [5]. Consequently, nutritional interventions targeting gut microbiota, including dietary bioactive compounds, have attracted considerable attention as potential strategies for improving obesity-associated metabolic disorders and hepatic steatosis [9,10,11].

Lutein, one of the dietary xanthophyll-type carotenoids, exhibits potent antioxidative and anti-inflammatory biological functions [12,13]. A body of research reveals that suppress inflammatory responses through the activation of the Nrf2 antioxidant pathway and suppression of NF-κB signaling cascades [14,15]. These properties position lutein as a promising candidate for modulating oxidative stress-mediated inflammatory diseases, including those of metabolic origin [1]. However, although these studies have demonstrated beneficial effects of lutein on individual aspects of metabolic regulation, relatively few investigations have comprehensively evaluated its simultaneous effects on hepatic histopathology, oxidative status, serum lipid metabolism, and gut microbiota composition in the same HFD-induced obesity model.

Therefore, this study was designed to investigate the effects of lutein supplementation on liver health, oxidative metabolism, serum lipid profiles, and gut microbiota composition. In addition, two supplementation doses were compared to evaluate potential dose-related differences. We hypothesized that lutein supplementation would attenuate HFD-induced metabolic disturbances and hepatic injury, and that these beneficial effects would be accompanied by improvements in antioxidant status and alterations in gut microbiota composition.

## 2. Materials and Methods

### 2.1. Reagents and Feeds

Lutein (S24939, chromatographic purity > 98%) was obtained from Shanghai Yuanye Biotechnology Co., Ltd. (Shanghai, China). Experimental feeds were acquired from Shanghai Shuyu Biotechnology Co., Ltd. (Shanghai, China). The AIN-93G standard formula was adopted: two experimental diets were used, a HFD with fat contributing 60% of total energy, and a low-fat diet (LFD) with fat contributing 10% of total energy. The main sources of fat were lard and soybean oil, and enough cholesterol. The two experimental diets were formulated to have equal total metabolizable energy (4021 kcal per formulation) by adjusting ingredient quantities, while the fat-to-carbohydrate energy contribution ratios differed (60% vs. 10% from fat). Consequently, the energy density of the HFD was 5.20 kcal/g, compared with 3.81 kcal/g for the LFD (Table 1). In each of the two diets, protein contributes 20% of the overall energy intake.

### 2.2. Animal and Experimental Design

The animal experimental protocols were examined and endorsed by the Institutional Animal Ethics Committee of the Modern Facilities Fisheries Research Institute (Approval Number: 202403018; Approval Date: 11 March 2024). Thirty-two male C57BL/6J mice (6 weeks old) were procured from Shandong Aimao Dakang Life Science Co., Ltd. (Weifang, China). After a 10-day acclimatization period with free access to a low-fat diet (LFD) and water (baseline 19.0 ± 2.0 g), four groups of mice (*n* = 8 each) were established through random assignment: (1) Ctrl group (LFD + vehicle, 0.5% CMC Na solution). (2) Mod group (HFD + vehicle). (3) Lutein-H group (HFD + 100 mg/kg/day lutein). (4) Lutein-L group (HFD + 50 mg/kg/day lutein). Lutein was freshly dispersed in 0.5% CMC-Na (carboxymethyl cellulose sodium) vehicle under light-protected conditions and administered once daily by oral gavage at a fixed volume of 10 μL/g body weight. A controlled housing environment (22–25 °C, 50–55% RH, 12 h light/dark) was used for all mice, with daily monitoring of body weight and feed intake. Feed consumption was calculated per cage and averaged to obtain group-level daily intake. Lutein was administered once daily for 70 consecutive days. All samples were collected at the terminal time point following 12 h fasting under identical experimental conditions. The doses of lutein (50 and 100 mg/kg/day) were selected based on previous studies reporting metabolic and antioxidant effects of lutein in rodents within the range of 20–150 mg/kg/day [16,17,18]. The 50 mg/kg/day dose was considered a moderate dose, while 100 mg/kg/day was used as a higher dose to evaluate potential dose–response effects [16]. Animal handling and sample processing staff were blinded for treatment assignments. No abnormal behavior or differences in feed intake were observed during the experimental period. All 8 mice per group were retained for body weight, liver weight and liver index measurement (*n* = 8). For hepatic biochemistry, serum lipid profiling and gut microbiota sequencing, 2 mice per group were excluded due to hemolysis and incomplete sample collection during dissection, resulting in *n* = 6 for subsequent detections.

### 2.3. Growth Performance Indices

Relative body weight gain (%) = (Final body weight/Initial body weight) × 100

Liver index (%) = [Liver wet weight (g)/Final body weight (g)] × 100

### 2.4. Biochemical Analyses

Blood samples were collected via the orbital sinus at sacrifice with isoflurane anesthesia. Mice were euthanized by cervical dislocation following anesthesia to minimize suffering in accordance with ethical guidelines. Blood was left to coagulate at ambient temperature for 30 min and then centrifuged at 3000 rpm for 10 min at 4 °C to collect serum, which was stored at −80 °C until analysis. Liver specimens were homogenized in ice-cold normal saline (1:9, *w*/*v*), followed by centrifugation at 3000 rpm for 10 min at 4 °C. The supernatant was harvested for biochemical assays. Superoxide dismutase (SOD), catalase (CAT), glutathione (GSH), malondialdehyde (MDA), alanine transaminase (ALT) and aspartate transaminase (AST) in liver homogenate, and serum total cholesterol (TC), triglycerides (TG), low-density lipoprotein cholesterol (LDL-C), high-density lipoprotein cholesterol (HDL-C) were determined using commercial assay kits obtained from Nanjing Jiancheng Bioengineering Institute (Nanjing, China). Prior to testing, liver homogenate supernatant was diluted with normal saline to fit each kit’s linear range (optimized via pre-experiment), with 2.5–20 μL loaded per microplate well. Undiluted serum (2.5 μL per well) was directly measured for TC, TG, LDL-C and HDL; serum for SOD, CAT, GSH and MDA was diluted appropriately before loading. Two quantification strategies were used as per kit instructions: (1) Gradient standard curves (R^2^ ≥ 0.99) for ALT, AST, SOD and CAT enzyme activity; sample values were calculated by substituting ΔA (A_sample − A_blank) into curve formulas and (2) single-point standard comparison for TC, TG, LDL-C, HDL, GSH and MDA, without serial standard gradients. All calculations followed manufacturer formulas, and mean absorbance of duplicate wells was used for analysis. Total protein concentration in liver supernatant was quantified with BCA kit (A045-4) for normalization of hepatic markers (per mg protein). Detailed kit specifications (catalog numbers, wavelengths, and principles) are as follows: SOD (A001-1-2, WST-1, 450 nm); CAT (A007-1-1, ammonium molybdate, 405 nm); GSH (A006-2-1, DTNB, 420 nm); MDA (A003-1-2, TBA, 532 nm); TC (A111-1-1, COD-PAP, 500 nm); TG (A110-1-1, GPO-PAP, 500 nm); LDL-C (A113-1-1) and HDL-C (A112-1-1) (selective masking enzymatic method, 550 nm); ALT (C009-2-1) and AST (C010-2-1) (2,4-dinitrophenylhydrazine colorimetry, 505 nm).

### 2.5. Gut Microbiota Analysis

Fecal samples were collected from the distal colon immediately following sacrifice on the final day of the 70-day intervention (i.e., the last day of treatment), prior to any other sample collection procedures. A total of six animals per group (*n* = 6) were used for gut microbiota sequencing. Samples were collected using sterile forceps and placed directly into sterile 2 mL Eppendorf tubes. To minimize contamination, all instruments were sterilized with 70% ethanol between samples, and collection was performed under a laminar flow hood. Immediately after collection, samples were snap-frozen in liquid nitrogen and subsequently stored at −80 °C until DNA extraction. The DNA extraction was performed within 3 weeks of sample collection for all samples simultaneously, using the EZNA Cycle-Pure Kit (Omega Bio-Tek, Inc., Norcross, GA, USA) following the manufacturer’s instructions. The V3–V4 hypervariable region of the bacterial 16S rRNA gene was amplified using universal primers 338F (5′-ACTCCTACGGGAGGCAGCAG-3′) and 806R (5′-GGACTACHVGGGTWTCTAAT-3′). The 25 μL polymerase chain reaction (PCR) amplification reaction mixture formulated as 12.5 μL KOD FX Neo high-fidelity DNA polymerase mix (TOYOBO, Osaka, Japan) containing 1 μL of forward primer (10 μM), 1 μL of reverse primer (10 μM), 1 μL of template DNA, and 9.5 μL of nuclease-free ddH_2_O. The following PCR conditions were used: initial denaturation at 94 °C for 3 min; then 30 cycles of (94 °C for 30 s, 56 °C for 45 s, 72 °C for 60 s); and a final extension at 72 °C for 10 min. PCR amplification products were purified using VAHTS DNA Clean Beads (Vazyme Biotech Co., Ltd., Nanjing, China), and sequencing libraries were generated with custom adapters via the VAHTS Universal V6 RNA-seq Library Prep Kit (Vazyme Biotech Co., Ltd., Nanjing, China). Library concentration and integrity were assessed using an Agilent 2100 Bioanalyzer (Agilent Technologies, Santa Clara, CA, USA) and Qsep-400 capillary electrophoresis system (BiOptic Inc., New Taipei City, Taiwan, China). Paired-end 150 bp (PE150) sequencing was performed on the DNBSEQ-T7 platform (MGI Tech Co., Ltd., Shenzhen, China). Raw paired-end reads were quality-filtered using Trimmomatic v0.33 with the following criteria: reads containing ambiguous N bases were discarded; trimming of low-scoring terminal nucleotides was implemented by adopting a 5 bp sliding window, where the average quality limit was set to Q20; sequences shorter than 150 bp after trimming were removed. Clean reads were denoised and processed in QIIME2 (v2020.6.0), and the SILVA 138.2 reference database was used for taxonomic classification. Indices of alpha diversity (Chao1, ACE, Shannon) and beta diversity based on Bray–Curtis distance were computed. Principal coordinate analysis (PCoA) combined with permutational multivariate analysis of variance (PERMANOVA; Adonis test) was applied to detect intergroup differences in overall microbial community composition. Linear discriminant analysis effect size (LEfSe) was applied to detect differential bacterial taxa among groups, with the multi-group comparison mode set to one-against-all and a linear discriminant analysis (LDA) score threshold > 3 for biomarker identification. Briefly, the Chao1 and ACE indices reflect community richness, estimating the number of taxa (species or operational taxonomic units, OTUs) within samples. The Shannon index represents alpha diversity by integrating both species richness and evenness, thereby reflecting the overall diversity and distribution of microbial communities. The LEfSe is a biomarker discovery algorithm that identifies taxa with statistically significant differences in relative abundance between groups and further estimates the effect size of each discriminative feature using LDA scores.

### 2.6. HE Staining

After sacrifice, liver tissues were rapidly dissected, and tissue blocks were cut from the identical region of the left hepatic lobe and fixed in 4% paraformaldehyde solution. After gradient ethanol dehydration and xylene clearing, tissues were embedded in paraffin wax and cut into 4–5 μm-thick sections using a paraffin microtome. Sections were mounted onto glass slides and baked at 60 °C for 2 h. For histological staining, sections of paraffin were fully dewaxed with xylene and then rehydrated via a descending ethanol series into distilled water. Slides were stained with hematoxylin solution for nuclear labeling, differentiated with 1% hydrochloric acid–alcohol solution, and blued with weak ammonia water. Subsequently, cytoplasmic counterstaining was performed using eosin solution. After gradient ethanol dehydration and xylene transparentization, neutral balsam was used to seal the sections. Histological examination and image capture were conducted under an optical microscope at 40× and 200× magnification.

### 2.7. Statistical Analysis

Statistical analyses were performed using SPSS 25.0 (Chicago, IL, USA). Data are presented as mean ± SD. Multi-group comparisons were conducted by one-way ANOVA, followed by Duncan’s post hoc test for pairwise comparisons. Statistical significance was set at *p* < 0.05. Figures were generated using GraphPad Prism 10.0 (San Diego, CA, USA).

## 3. Results

### 3.1. Effects of Lutein on Body Weight, Hepatic Weight, and Hepatic Index

**Figure 1:**
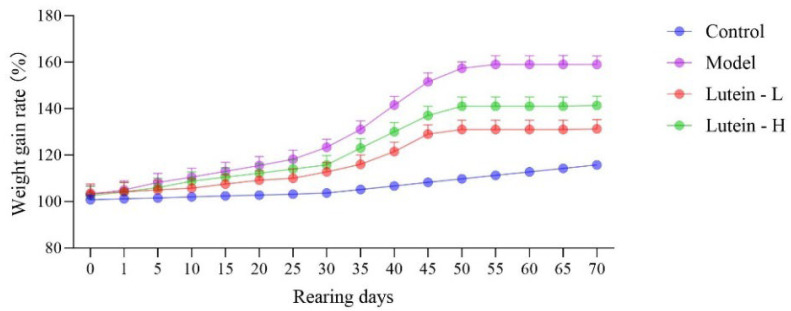
Changes in body weight of mice in different experimental groups. Sampling was conducted at 4-day intervals, with 8 mice per group (*n* = 8). All data were expressed as mean ± SD.

Daily feed intake was similar among the treatment groups during the experimental period (*p* > 0.05; Table 2). There was a significant increase in final body weight in the Mod group relative to the Ctrl group (Figure 1). Both low- and high-dose lutein treatments reduced body weight compared with the Mod group (*p* < 0.05); however, the Lutein-L group was not different from the Ctrl group, whereas the Lutein-H group remained significantly heavier than the Ctrl group. Compared with the Mod group liver index fell by 14.2% in the Lutein-L group and 26.1% in the Lutein-H group, with both reductions reaching statistical significance (*p* < 0.05), and the high-dose effect being more pronounced. Absolute liver weight in the Mod group was significantly elevated relative to that in all other groups, while no differences were observed among Ctrl, Lutein-L and Lutein-H groups (*p* > 0.05).

### 3.2. Hepatic Antioxidant Enzyme Activity and Oxidative Stress Indicators

Compared with the Ctrl group, the Mod group showed reduced GSH content and elevated MDA levels (*p* < 0.05; Table 3), while SOD and CAT activities showed no significant changes (*p* > 0.05). Compared with the Mod group, lutein supplementation altered hepatic oxidative biomarkers toward the Ctrl phenotype. The Lutein-H group exhibited a 28.11% increase in SOD activity and a 44.19% increase in CAT activity (*p* < 0.05). Relative to the Mod group, GSH content increased by 75.82% in the Lutein-L group and 204.95% in the Lutein-H group (*p* < 0.05). MDA levels decreased by 56.68% in Lutein-L and 58.29% in Lutein-H compared with the Mod group (*p* < 0.05).

### 3.3. Serum Lipid Profiles and Hepatic Injury Markers

Compared to the Ctrl group, the Mod group showed higher TG, T-CHO, LDL-C, ALT and AST levels, accompanied by reduced HDL-C (*p* < 0.05; Table 4). Compared to the Mod group, both lutein-treated groups exhibited significantly higher HDL-C levels (*p* < 0.05). Serum LDL-C was significantly decreased only in the Lutein-H group, while the Lutein-L group showed a non-significant reduction. Serum TG levels were not altered by either dose (*p* > 0.05). Serum ALT and AST activities were decreased in both lutein-treated groups, with greater changes observed in the Lutein-H group.

### 3.4. Gut Microbiota Composition

**Figure 2:**
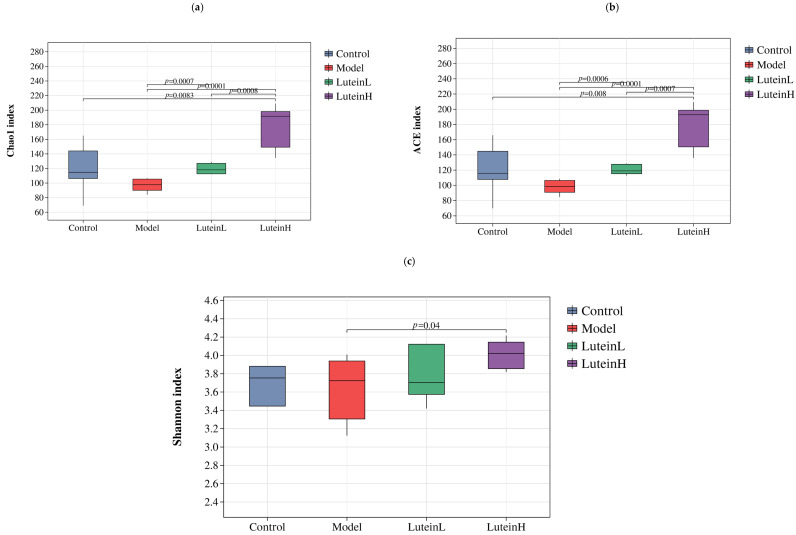

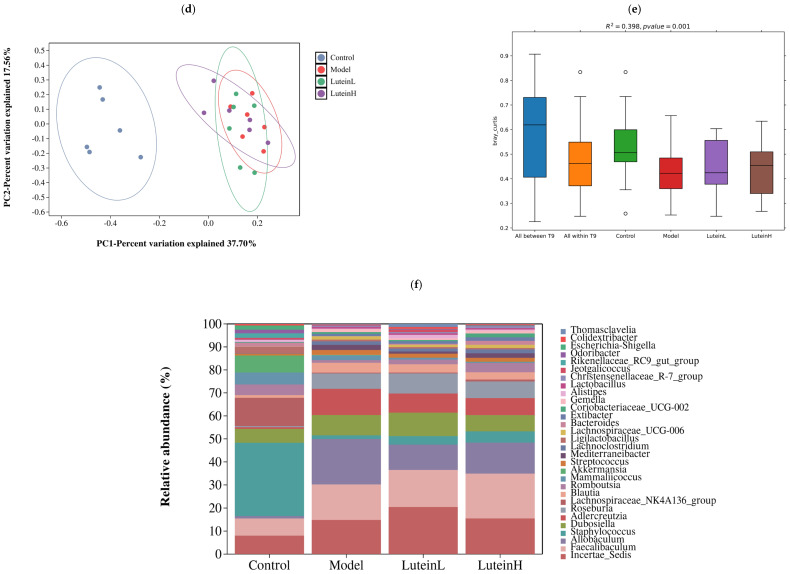

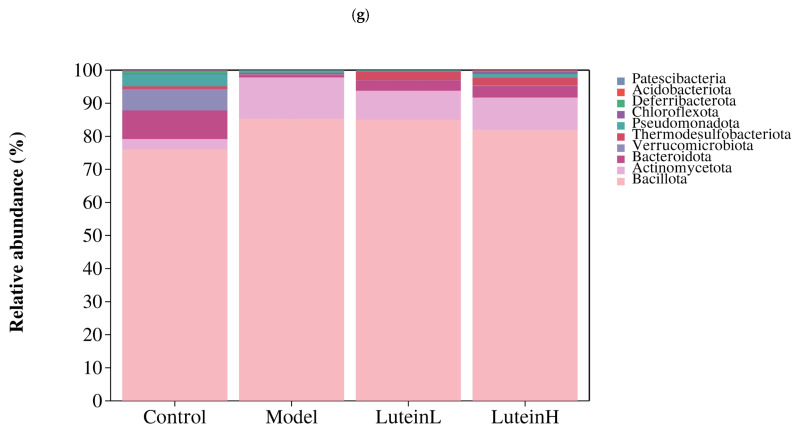

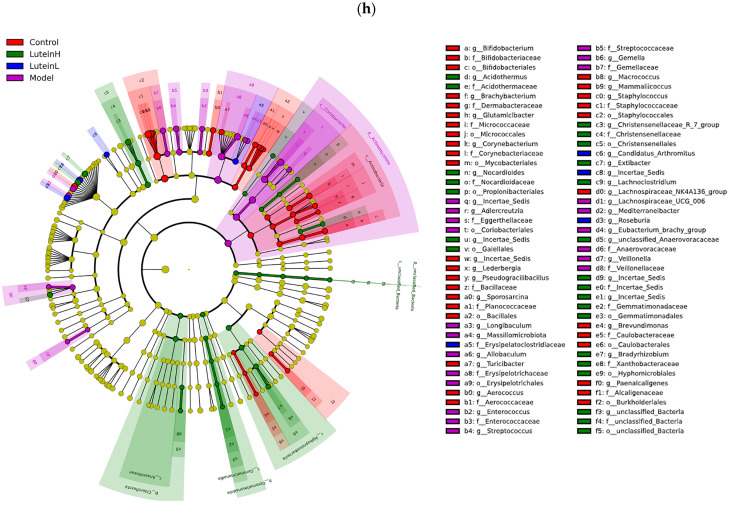

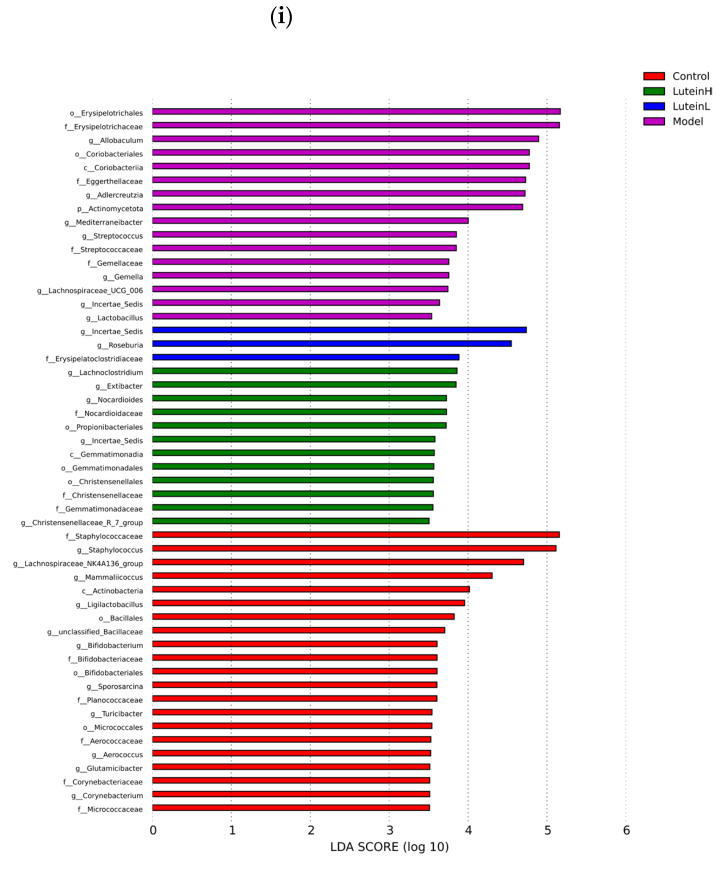
Effects of Lutein Intervention on gut microbiome. (**a**–**c**) Alpha diversity index (**a**) Chao1 Index (**b**), ACE Index, (**c**) Shannon Index. One-way ANOVA was applied for intergroup comparisons, followed by Duncan’s multiple range test for post hoc correction of multiple comparisons. (**d**) PCoA of intestinal microbiota structure across different experimental groups. This PCoA plot (Bray–Curtis distance, Adonis intergroup test) shows sample community similarity/differences; each group’s 95% confidence ellipse is included. (**e**) Comparison of Bray–Curtis dissimilarities among and within experimental groups. Statistical significance was assessed by PERMANOVA (R^2^ = 0.398, *p* = 0.001). (**f**) Genus-level variations in gut microbiota structure. (**g**) Phylum-level variations in gut microbiota structure. Highlighted genera. (**h**) presents cladograms depicting the evolutionary branching of distinct bacterial taxa with differential distribution. (**i**) displays linear discriminant analysis (LDA) scores from LEfSe analysis, with the threshold set at LDA score > 3 (log10-scaled).

Lutein-H significantly increased alpha diversity indices (ACE, Chao1, and Shannon) compared with the Mod group (Figure 2a–c), indicating an improvement in gut microbial richness and diversity following high-dose intervention. In the principal coordinates analysis (PCoA) based on Bray–Curtis distances, the Ctrl group was clearly separated from the Mod group, reflecting distinct microbial community structures. The lutein-treated groups partially overlapped with the Mod group but exhibited a clear shift toward the Ctrl group, with the Lutein-H group showing the most pronounced restoration effect (Figure 2d). Significant intergroup variations in global microbial community composition were identified through PERMANOVA. (R^2^ = 0.398, *p* = 0.001) (Figure 2e). At the genus level, Lutein-H enriched beneficial taxa, including *Roseburia* and *Faecalibaculum* (Figure 2f). At the phylum level, HFD-induced reduction in *Bacteroidota* abundance was largely restored in the Lutein-H group (Figure 2g). LEfSe analysis further identified distinct bacterial biomarkers associated with the Lutein-H group, as shown in the cladogram and LDA score plots (Figure 2h,i), including enrichment of *Lachnospiraceae* and *Clostridia_vadinBB60* (LDA > 3).

### 3.5. Histomorphology

**Figure 3:**
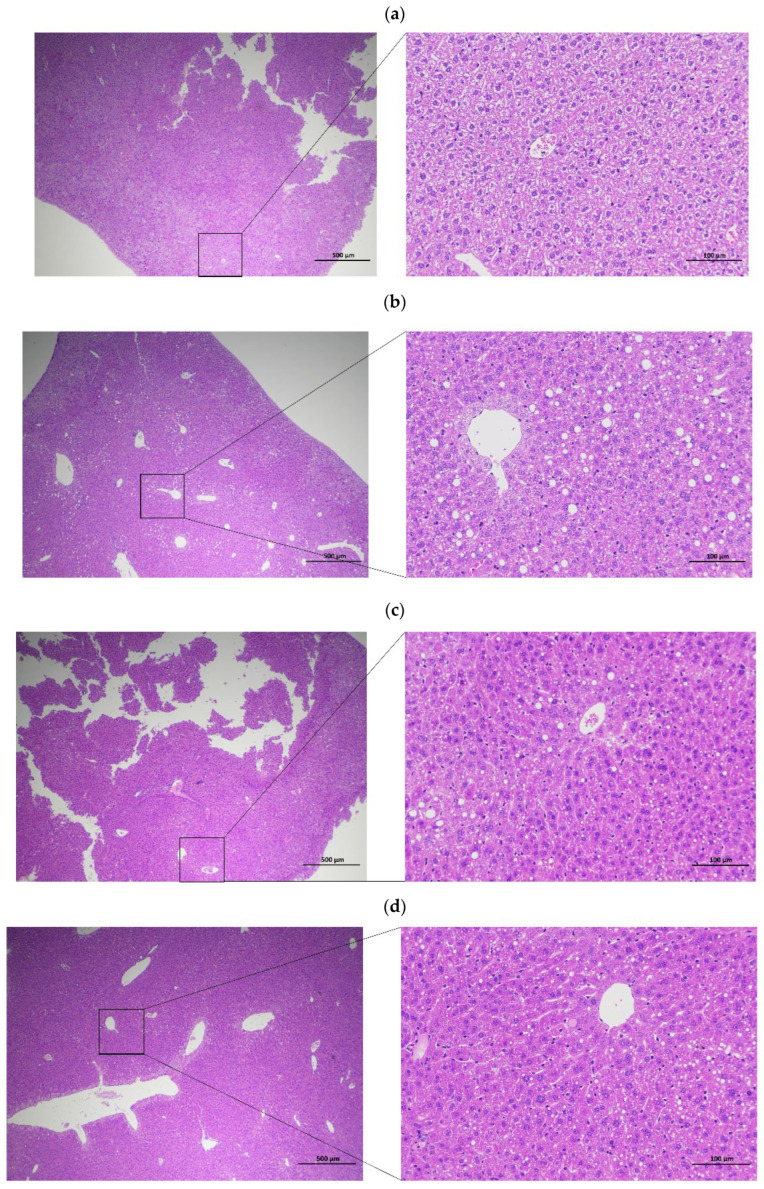
Results of HE staining of liver tissue sections in each experimental group. (**a**) Ctrl. (**b**) Mod. (**c**) Lutein-L. (**d**) Lutein-H. Magnification: 40× (**left**) and 200× (**right**).

The Lutein-H group showed reduced hepatocellular vacuolation, and improved hepatic lobule structure (Figure 3). The Lutein-L group showed reduced vacuole size and number but barely restored liver structure, while the Mod group exhibited extensive vacuolation, disordered hepatic cords. The histological changes were further quantified using the NAFLD Activity Score (NAS) among groups (Table 5). The Mod group presented a total NAS of 4, while the Lutein-L group decreased to three (moderate alleviation) and the Lutein-H group reached one, close to the Ctrl group (NAS = 0).

## 4. Discussion

The present study was planned to comprehensively evaluate the effects of lutein supplementation on liver health, oxidative status, lipid metabolism, and gut microbiota in high-fat diet fed mice, and the findings collectively demonstrate its beneficial metabolic and hepatoprotective effects. Daily feed intake was comparable across all groups during the intervention, suggesting that differences in feed consumption were unlikely to confound the observed changes in body weight and liver morphology [16]. The HFD-induced body weight gain in the Mod group, whereas lutein supplementation at both doses attenuated this increase. The liver index, a commonly used indicator of HFD-induced hepatomegaly and lipid accumulation, was decreased by lutein in a dose-dependent fashion with both low- and high-dose groups showing reductions compared to the Mod group, and a more marked effect observed at the higher dose, consistent with carotenoid-associated hepatoprotective effects reported in models of fatty liver disease [19]. This dose-dependent trend suggests greater efficacy at the higher dose, while the low dose still elicited a statistically significant effect on liver index. Notably, absolute liver weight in lutein-treated groups approached the levels observed in the Ctrl group.

Lutein supplementation markedly improved the hepatic antioxidant capacity in HFD-fed mice. Specifically, mice in the Lutein-H group showed elevated SOD and CAT activities in comparison with the Mod group. Similarly, higher GSH content and reduced MDA levels were found in both lutein-supplemented groups (Lutein-L and Lutein-H) relative to the Mod group. These divergent dose–response patterns among antioxidant biomarkers may reflect differences in their sensitivity to lutein exposure. MDA reduction appears to reach a plateau at lower doses, whereas the restoration of antioxidant enzymes and GSH levels may require higher levels of lutein supplementation. These findings are consistent with previous studies showing that lutein reduces oxidative stress, limits lipid peroxidation, and decreases lipid accumulation in hepatic cells, reflecting the well-established antioxidant properties of carotenoids [20,21,22]. Notably, lutein-derived fragments may demonstrate more potent antioxidant and anti-inflammatory properties than the parent compound [23].

Lutein has been demonstrated to prevent alcohol-induced liver damage through modulating inflammatory responses and oxidative stress [24,25], decrease oxidative stress in hypercholesterolemic guinea pigs [26], and protect against chemically induced hepatic injury [27]. Consistent with these observations, the improved hepatic antioxidant status observed in the present study further supports the hepatoprotective potential of lutein, possibly through improvement of endogenous antioxidant defense and improvement of hepatic redox homeostasis in HFD-induced mice [28,29,30]. Notably, hepatic GSH and MDA showed different responses to lutein supplementation with MDA levels restored to near-Ctrl values in both groups, whereas GSH recovery was observed only at the high dose. The MDA, a terminal product of lipid peroxidation generated through a series of ROS-mediated oxidative reactions [31], accumulated only in the HFD model group, whereas both lutein supplementation groups maintained MDA levels comparable to the control group without significant dose-dependent differences. This pattern may be explained by the fact that inhibition of early oxidative events can prevent the subsequent propagation of lipid peroxidation and reduce the formation of MDA precursors [32]. Moreover, as MDA reflects accumulated oxidative damage, it may be less sensitive than upstream antioxidant defense markers such as SOD, CAT, and GSH in distinguishing subtle dose-related responses [33]. Consistent with this view, antioxidant biomarkers do not always change in parallel under oxidative stress challenges [34]. Therefore, the similar MDA levels observed in Lutein-L and Lutein-H groups do not contradict the dose-dependent changes in antioxidant enzyme activities. This discrepancy may reflect their distinct biological properties, as MDA represents product of lipid peroxidation, while GSH is dynamic intercellular antioxidant pool dependent on synthesis and recycling [35]. In addition, because antioxidant biomarkers often respond differently during nutritional interventions [36,37], the present findings suggest that low-dose lutein supplementation may be sufficient to attenuate lipid peroxidation, whereas higher doses may be more effective in supporting the restoration of intracellular glutathione homeostasis.

Hepatic SOD and CAT activities increased with high lutein supplementation, which seemingly conflicts with the classical low-dose adaptive response and hormetic model. However, hormesis is defined as a biphasic dose–response phenomenon triggered by mild oxidative or chemical stress that drives compensatory antioxidant activation [38]. This mode of action fundamentally differs from non-toxic dietary antioxidants such as lutein. Unlike oxidative stressors, carotenoids exert dual functions via direct ROS scavenging and modulation of intracellular redox signaling networks [32]. Previous hepatic research has confirmed that lutein activates Nrf2 cascades to upregulate downstream antioxidant enzymes including SOD and CAT [39], and the biological potency of carotenoids generally correlates with their tissue concentration within safe effective ranges [22]. Within the 50–100 mg/kg/day lutein dosage range, the high-dose group achieved greater hepatic lutein bioavailability, likely further amplified Nrf2-mediated antioxidant defense [40] and yielded higher SOD and CAT activity. Collectively, the Lutein-H group cannot be regarded as a mild low-level stress stimulus; the elevated antioxidant enzyme activities observed reflect dose-strengthened antioxidant capacity rather than just a stress-triggered adaptive feedback.

Consistently, lutein supplementation was associated with significant improvements in serum lipid profiles, including elevated HDL-cholesterol levels (in both doses) and decreased LDL-cholesterol (only at the high dose), as well as alleviated liver injury reflected by reduced serum ALT and AST activities. Overall, serum lipid indicators exhibited heterogeneous responses across dose groups. Lutein groups exhibited significantly elevated HDL-C levels relative to the Mod group, but only the low-dose group showed a significant increase relative to both Mod and Ctrl, while the high-dose group was not different from Ctrl. The ALT/AST levels showed a trend toward gradual decrease with increasing lutein exposure. These metabolic benefits are consistent with clinical observations where lutein supplementation reduced plasma lipid peroxidation and inflammatory markers [41] and align with studies highlighting its anti-obesity and lipid-lowering potential [17,42,43]. Previous studies have observed a correlation between lutein intervention and relieved non-alcoholic fatty liver disease symptoms [44], and its therapeutic potential in steatosis liver disease continues to be explored [19]. In contrast to prior rodent studies [45], serum TG concentrations were not significantly affected by either low- or high-dose lutein intervention in HFD-fed mice. However, total cholesterol (TC) showed distinct dose-dependent responses: TC levels of the low-dose lutein group were comparable to the Mod group without significant improvement, whereas the high-dose group exhibited a marked reduction in TC, which aligns with a human meta-analysis reporting inconsistent effects of lutein supplementation on circulating TC levels in clinical populations [46]. The LDL-C was significantly decreased in the Lutein-H group relative to the Mod group, whereas the Lutein-L group showed a non-significant reduction. The different responses between TG and cholesterol-related lipids may be attributed to interspecies differences and metabolic context variations between HFD-induced obese mice and human subjects. Interestingly, HDL-C was increased in the Lutein-L group, relative to both the Ctrl and Lutein-H groups, suggesting a possible dose-dependent regulatory effect of lutein on HDL metabolism in mice [45]. Regarding liver function markers, serum ALT and AST showed a decreasing trend in a dose-dependent manner after lutein supplementation, although levels did not fully return to those of the Ctrl group. This indicates that lutein may be associated with partial improvement of HFD-induced hepatic injury, while complete recovery may require longer treatment duration or different dosing strategies [47,48].

A novel aspect of our study is the observed modulation of gut microbiota by lutein, which may serve as a potential correlative factor for its systemic metabolic benefits. Notably, beneficial microbial alterations were mainly observed in the high-dose lutein group, which may reflect a dose-dependent response in gut microbiota changes. The high-dose lutein group (Lutein-H) exhibited higher microbial alpha diversity (Chao1, ACE, and Shannon indices) and relative enrichment of beneficial genera such as *Roseburia* and *Faecalibaculum*. This reshaping of the gut ecosystem resonates with established links between gut microbiome richness and metabolic health [49] and the known alterations in gut microbial ecology in obesity [50]. The increase in *Roseburia*, a butyrate-producing genus, is consistent with previous observations that carotenoids can modulate gut microbiota composition and promote the growth of short-chain fatty acid-producing bacteria, including *Roseburia* [51]. The partial restoration of the phylum *Bacteroidota*, which is often depleted in NAFLD [52] may be associated with metabolic improvements, although causal relationships cannot be inferred from the present study.

The observed microbial shifts are comparable to those induced by other dietary interventions. Dietary fat composition significantly impacts gut microbiota and cardiometabolic risk [53,54]. Similarly, probiotics such as *Lactobacillus rhamnosus* [55] and *Lactobacillus plantarum* [56] alleviate HFD-induced obesity by modulating microbial communities. Other dietary components, including Fuzhuan brick tea [57] and wheat bran oil [58], also exert metabolic benefits through gut microbiota remodeling. The enrichment of Christensenellaceae_R-7 group in the Lutein-H group is consistent with microbial signatures associated with leanness identified in machine learning studies [59]. Furthermore, the changes in the relative abundance of potentially pro-inflammatory taxa were also observed in gut microbiota phenotypes associated with obesity [60] and in rodent models of metabolic disease [61].

The high-dose lutein group showed greater improvements in certain parameters (e.g., liver index, antioxidant enzymes, TC and LDL-C), whereas the low-dose group exhibited better effects on body weight and HDL-C. Consequently, not all measured parameters displayed a clear dose-dependent pattern. This observation may reflect the complex biological behavior of lutein in vivo. As a highly lipophilic carotenoid, lutein absorption has been suggested to depend on micellar solubilization and transporter-mediated uptake, processes that may become less efficient at higher intake levels and therefore may not lead to proportional increases in tissue exposure [62,63]. In addition, the relatively low and variable bioavailability of lutein, together with differences in absorption, distribution, and metabolism among individual animals, may also have contributed to variability in the observed responses [62,64]. Moreover, antioxidant regulation and gut microbial homeostasis involve multiple interacting physiological processes, which may respond differently to increasing doses of dietary bioactive compounds. Collectively, these multi-faceted mechanisms may underlie the heterogeneous dose–response patterns observed across hepatic, serum, and intestinal endpoints. The overall trend toward greater efficacy in the high-dose group, together with the absence of a consistent dose–response relationship across all indicators needs further exploration.

This study has the limitations of relatively small sample size and the exclusive use of male mice. In addition to the interspecies differences, glucose metabolism, inflammatory biomarkers, short-chain fatty acid measurements, molecular and antioxidant pathways, mechanisms at the level of gene expression, and direct hepatic and intestinal inflammmation were not measured. Furthermore, the observed alterations in gut microbiota were correlative, and the causal relationship between microbial remodeling and the hepatoprotective effects of lutein warrants further investigation. Another limitation is that only two lutein doses (50 and 100 mg/kg/day; 2-fold difference) were evaluated without a low-dose group for full concentration gradient. Building on these preliminary findings, future studies should include a broader dose range to determine the optimal dosage of lutein.

Overall, these results support the potential value of lutein as a dietary component for preventing or managing obesity-related liver disorders and gut dysbiosis. Therefore, dietary lutein supplementation could be developed as a promising nutritional approach to ameliorate obesity-associated pathologies. However, the doses employed in the current study (50 and 100 mg/kg/day), particularly the high-dose regimen (100 mg/kg/day), are relatively high in the context of experimental settings and may not directly reflect usual human dietary intake levels (approximately 1–3 mg/day based on dietary intake surveys) [65,66]. This comparison highlights that the experimental doses represent pharmacological exposure rather than habitual dietary consumption. Nevertheless, such doses are commonly employed in preclinical studies to elucidate biological mechanisms and therapeutic potential. To facilitate translation of these findings to human populations, further studies applying body surface area-based allometric scaling approaches [67,68] and evaluating nutritionally relevant doses will help establish physiologically appropriate intake ranges as interspecies dose translation is not linear.

## 5. Conclusions

Supplementation with lutein, at the high dose (100 mg/kg/day), in particular, which exerted significant protective effects on HFD-fed mice compared to the Mod group, specifically in terms of liver health, lipid metabolism, and gut microbiota homeostasis. It effectively ameliorated hepatic steatosis, improved systemic antioxidant capacity, partially improved serum lipid profiles (notably TC, LDL-C, and HDL-C, though TG remained unchanged), and beneficially reshaped the gut microbiota structure. Although the high-dose group generally exhibited greater effects than the low-dose group, responses were not uniformly dose-dependent across all measured parameters, suggesting complex pharmacokinetic and biological characteristics of lutein. Further studies are warranted to elucidate its absorption and metabolism, determine optimal supplementation levels in human, and assess its efficacy and safety for potential clinical translation.

## Figures and Tables

**Table 1:** Ingredient composition of the experimental diet and corresponding energy contribution.

	HFD (60% kcal Fat)		LFD (10% kcal Fat)	
**Macronutrient ^a^**	**Weight** **Proportion (%)**	**Energy** **Contribution (%)**	**Weight** **Proportion (%)**	**Energy** **Contribution (%)**
Protein	26	20	19.2	20
Carbohydrate	26	20	67.3	70
Fat	35	60	4.3	10
Total		100		100
**Ingredient ^b^**	**Weight (g)**	**Energy contribution (kcal)**	**Weight (g)**	**Energy contribution (kcal)**
Casein	200	800	200	800
L-cystine	3	12	3	12
Corn starch	0	0	506.2	2024.8
Maltodextrin	125	500	125	500
Sucrose	72.8	275	72.8	275
Cellulose	50	0	50	0
Soybean oil	25	225	25	225
Lard	245	2205	20	180
Mineral mix	50	0	50	0
Vitamin mix	1	4	1	4
Choline bitartrate	2	0	2	0
Total		4021		4021

^a^ Macronutrient energy contribution (%) was calculated using standard Atwater factors (protein and carbohydrate: 4 kcal/g; fat: 9 kcal/g). ^b^ Ingredient weights are expressed in grams per diet formulation, and energy values (kcal) were calculated based on the caloric content of each ingredient. Non-caloric components were included in the formulation but excluded from energy calculations.

**Table 2:** Effects of lutein on feed intake, body weight, liver weight and liver index in mice.

Group	Average Daily Feed Intake (g)	Body Weight (g)	Liver Weight (g)	Liver Index (%)
Ctrl	15.62 ± 1.12	21.96 ± 1.37 ^c^	0.86 ± 0.12 ^b^	3.91 ± 0.41 ^a^
Mod	15.73 ± 1.35	30.29 ± 3.94 ^a^	1.19 ± 0.15 ^a^	3.95 ± 0.25 ^a^
Lutein-L	15.42 ± 1.03	24.94 ± 3.78 ^bc^	0.83 ± 0.10 ^b^	3.39 ± 0.54 ^b^
Lutein-H	15.56 ± 1.17	26.86 ± 1.96 ^b^	0.78 ± 0.10 ^b^	2.92 ± 0.33 ^c^

Values are reported as mean ± SD (*n* = 8). Dissimilar superscript letters denote statistically significant discrepancies between groups (*p* < 0.05).

**Table 3:** Hepatic antioxidant enzyme activities and oxidative stress-related biomarkers.

Group	SOD (U/mg prot.)	CAT (U/mg prot.)	GSH (μmol/g prot.)	MDA (nmol/mg prot.)
Ctrl	128.64 ±14.51 ^ab^	60.43 ± 3.64 ^b^	5.60 ± 0.59 ^a^	0.78 ± 0.18 ^b^
Mod	120.59 ± 33.55 ^b^	65.36 ± 3.50 ^b^	1.82 ± 0.42 ^c^	1.87 ± 0.60 ^a^
Lutein-L	135.17 ± 15.08 ^ab^	87.80 ± 13.52 ^a^	3.20 ± 0.49 ^b^	0.81 ± 0.21 ^b^
Lutein-H	154.49 ± 10.53 ^a^	94.24 ± 13.06 ^a^	5.55 ± 0.76 ^a^	0.78 ± 0.10 ^b^

Values are reported as mean ± SD (*n* = 6). Dissimilar superscript letters denote statistically significant discrepancies between groups (*p* < 0.05).

**Table 4:** Contents of blood lipid indicators and liver injury markers among different lutein dose groups.

Group	TG (mmol/L)	T-CHO (mmol/L)	HDL-C (mmol/L)	LDL-C (mmol/L)	ALT (U/L)	AST (U/L)
Ctrl	1.65 ± 0.55 ^a^	1.62 ± 0.32 ^c^	2.44 ± 0.43 ^bc^	0.11 ± 0.01 ^c^	18.67 ± 5.20 ^d^	27.69 ± 5.08 ^d^
Mod	1.92 ± 0.48 ^ab^	2.98 ± 0.45 ^a^	2.15 ± 0.19 ^c^	0.26 ± 0.05 ^a^	80.87 ± 9.99 ^a^	117.82 ± 16.09 ^a^
Lutein-L	1.16 ± 0.20 ^b^	2.95 ± 0.43 ^a^	2.93 ± 0.19 ^a^	0.24 ± 0.04 ^ab^	54.15 ± 2.24 ^b^	69.58 ± 14.95 ^b^
Lutein-H	1.34 ± 0.41 ^b^	2.30 ± 0.48 ^b^	2.57 ± 0.17 ^b^	0.20 ± 0.03 ^b^	33.45 ± 7.70 ^c^	42.29 ± 7.99 ^c^

Values are reported as mean ± SD (*n* = 6). Dissimilar superscript letters denote statistically significant discrepancies between groups (*p* < 0.05).

**Table 5:** Histological evaluation of liver tissues using NAFLD Activity Score (NAS).

Group	Steatosis Score	Lobular Inflammation	Ballooning	NAS
Ctrl	0	0	0	0
Mod	2	1	1	4
Lutein-L	1	1	1	3
Lutein-H	1	0	0	1

Histological evaluation was performed in a blinded manner on hematoxylin and eosin (H&E)-stained liver sections. NAFLD Activity Score (NAS) was assessed according to the scoring system proposed by Kleiner [2], including steatosis (0–3), lobular inflammation (0–3), and hepatocyte ballooning (0–2). The total NAS was obtained by summing the three individual components.

## Data Availability

The raw data supporting the conclusions of this article will be made available by the authors on request.
